# Case Report: Imaging Features of Gallbladder Sessile Polyp Confirmed by Contrast-Enhanced Ultrasonography and Dynamic Computed Tomography in a Dog With Asymptomatic Chronic Cholecystitis

**DOI:** 10.3389/fvets.2022.836414

**Published:** 2022-04-01

**Authors:** Jeongmin Lee, Jinsu Kang, Suyoung Heo, Kichang Lee, Hakyoung Yoon

**Affiliations:** ^1^Department of Veterinary Medical Imaging, College of Veterinary Medicine, Jeonbuk National University, Iksan-si, South Korea; ^2^Department of Surgery, College of Veterinary Medicine, Jeonbuk National University, Iksan-si, South Korea

**Keywords:** biliary system, canine, coprophagia, gallbladder polyp, ultrasound

## Abstract

A 6-year-old dog was presented for health screening. It never suffered from any disease. Ultrasonography confirmed mass-like thickening with irregular margins protruding toward the gallbladder (GB) lumen. On contrast-enhanced ultrasonography (CEUS) and dynamic computed tomography (CT), contrast enhancement of the corresponding structures was confirmed in arterial phase. After cholecystectomy, cauliflower-like sessile polyps were identified. Histopathological examination revealed chronic lymphoplasmacytic cholecystitis. Bile culture revealed *Escherichia coli* growth. Our novel findings suggest that chronic cholecystitis should be considered as a differential diagnosis if contrast-enhanced sessile polyps of the GB are found on CEUS and dynamic CT.

## Introduction

In veterinary medicine, there are few reports on the imaging features of bacterial cholecystitis (1, 2). Furthermore, a previous study showed that gallbladder (GB) polyps or mass can indicate neoplasia including GB adenoma or adenocarcinoma (3, 4); cholecystitis may also be involved (5). Therefore, although it is important to diagnose polyp in the GB, it is difficult to accurately distinguish it from wall-attached sludge only by ultrasonography. In this regard, there were studies using contrast-enhanced ultrasonography (CEUS) to diagnose GB polyp or mass (4, 5). However, based on our literature review, there are no reports that describe the imaging features of GB polyps using dynamic computed tomography (CT) and there are no reports to simultaneously describe CEUS and dynamic CT imaging features of chronic cholecystitis with sessile polyps of the dog. Therefore, we aimed to describe a case wherein GB polyp was diagnosed using CEUS and dynamic CT and we compared the advantages of the two modalities.

## Case Description

A 6-year-old, spayed female beagle dog was admitted to the Jeonbuk National University Animal Medical Center for health screening. It showed no clinical signs and had never suffered from any disease but practiced coprophagia. No abnormal findings were observed in blood chemistry test, but a complete blood count revealed leukocytopenia (3.5 × 10^9^ cells/L; reference range, 6–17 × 10^9^ cells/L).

**Figure 1:**
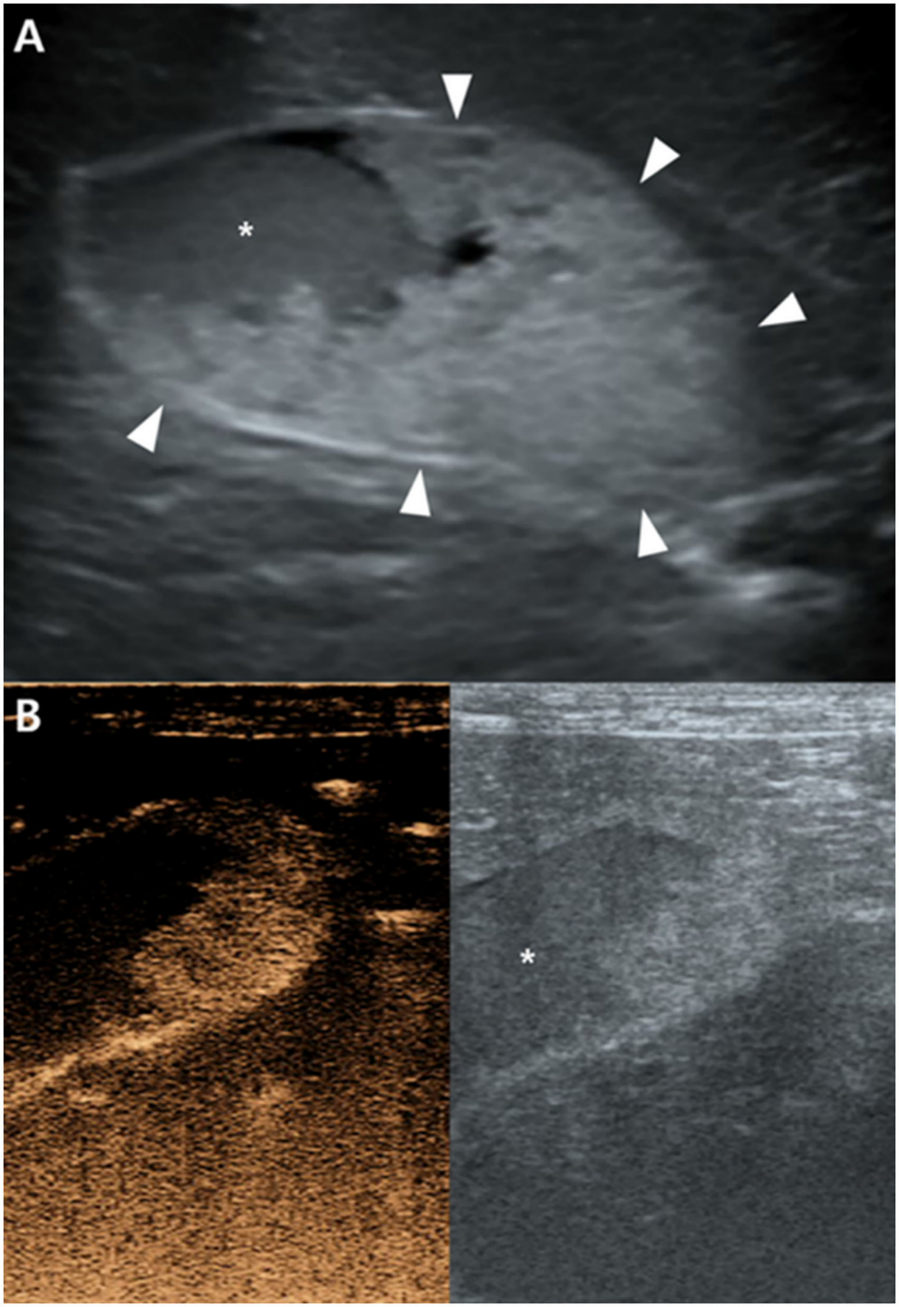
Ultrasonography **(A)** and contrast-enhanced ultrasonography (CEUS) **(B)** of the gallbladder (GB). **(A)** The hyperechoic structure (arrowheads) protruding toward the GB lumen appear more echogenic than GB contents (asterisk). **(B)** Strong CE of this structure is confirmed in CEUS 13 s after contrast medium injection. GB contents were not enhanced.

**Figure 2:**
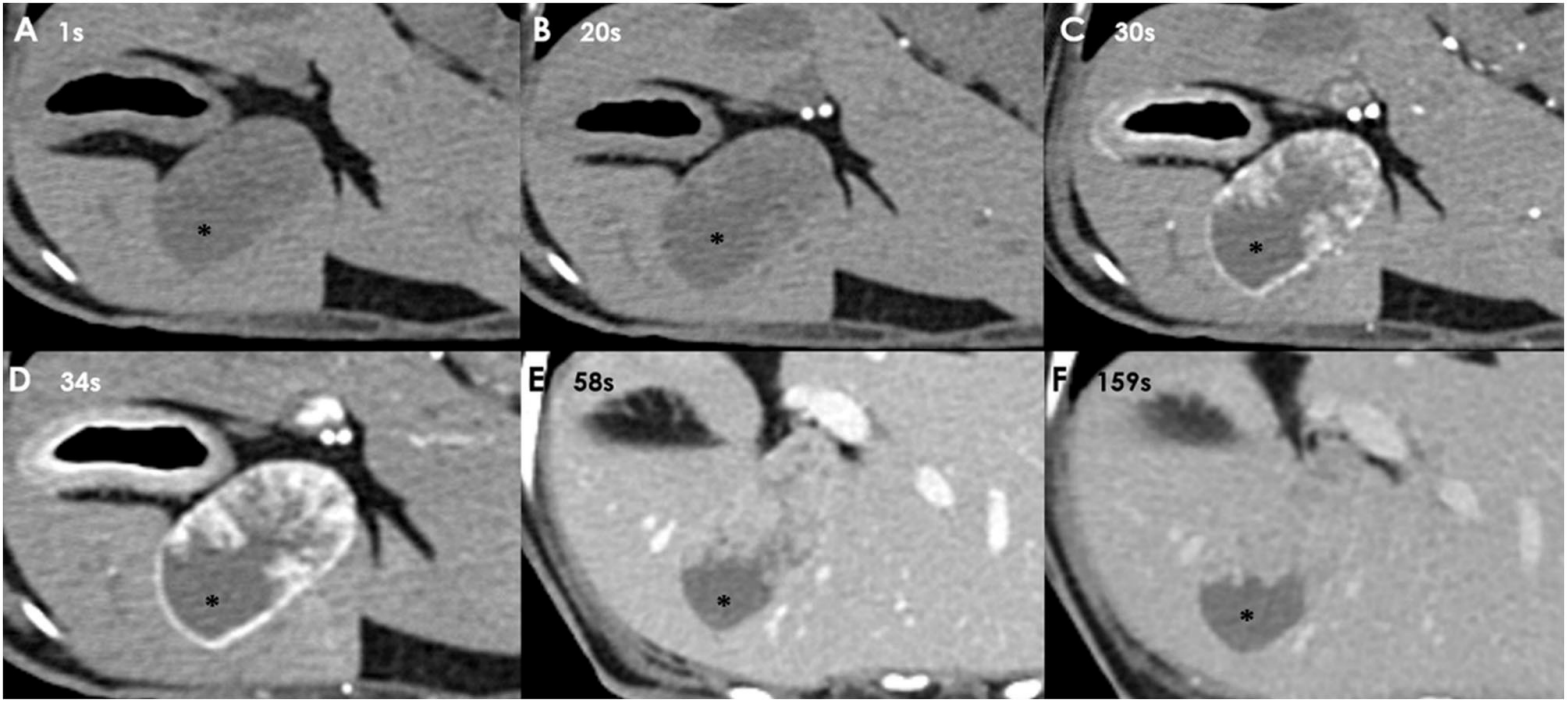
Dynamic CT images taken 1 s **(A)**, 20 s **(B)**, 30 s **(C)**, and 34 s **(D)** after CM injection. Portal phase image of the GB taken 58 s **(E)**, and delayed phase image of the GB taken 159 s **(F)** after CM injection. CE is observed in the protruding structures of the GB wall when more than 20 s have passed since CM injection. GB contents (asterisk) are not enhanced (HU: 20; arterial phase).

**Figure 3:**
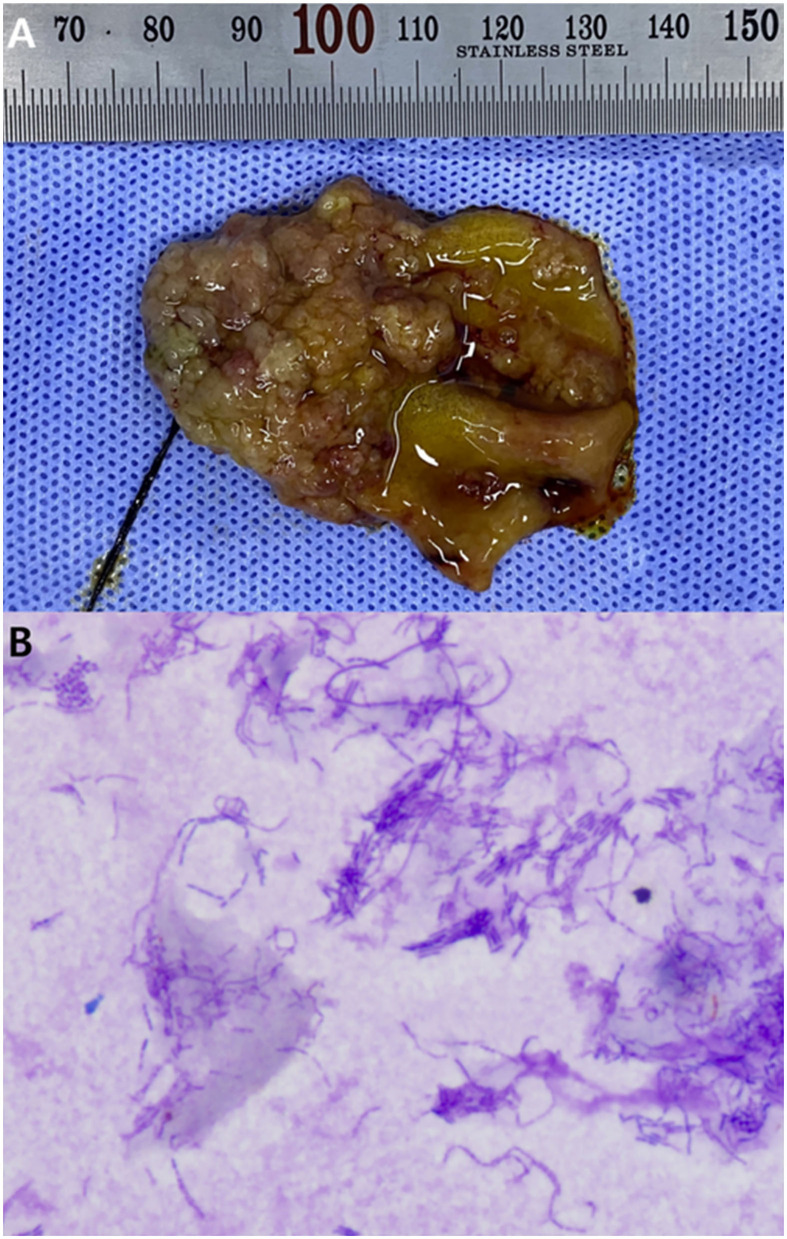
Gross lesion of the inner surface of the resected gall bladder **(A)**, and cytology of the bile **(B)**. Diff quick staining, 1,000 × magnification. **(A)** Cauliflower-like sessile polyps are observed. **(B)** A number of bacilli observed in the bile. Bile culture revealed these to be *E. coli*.

**Figure 4:**
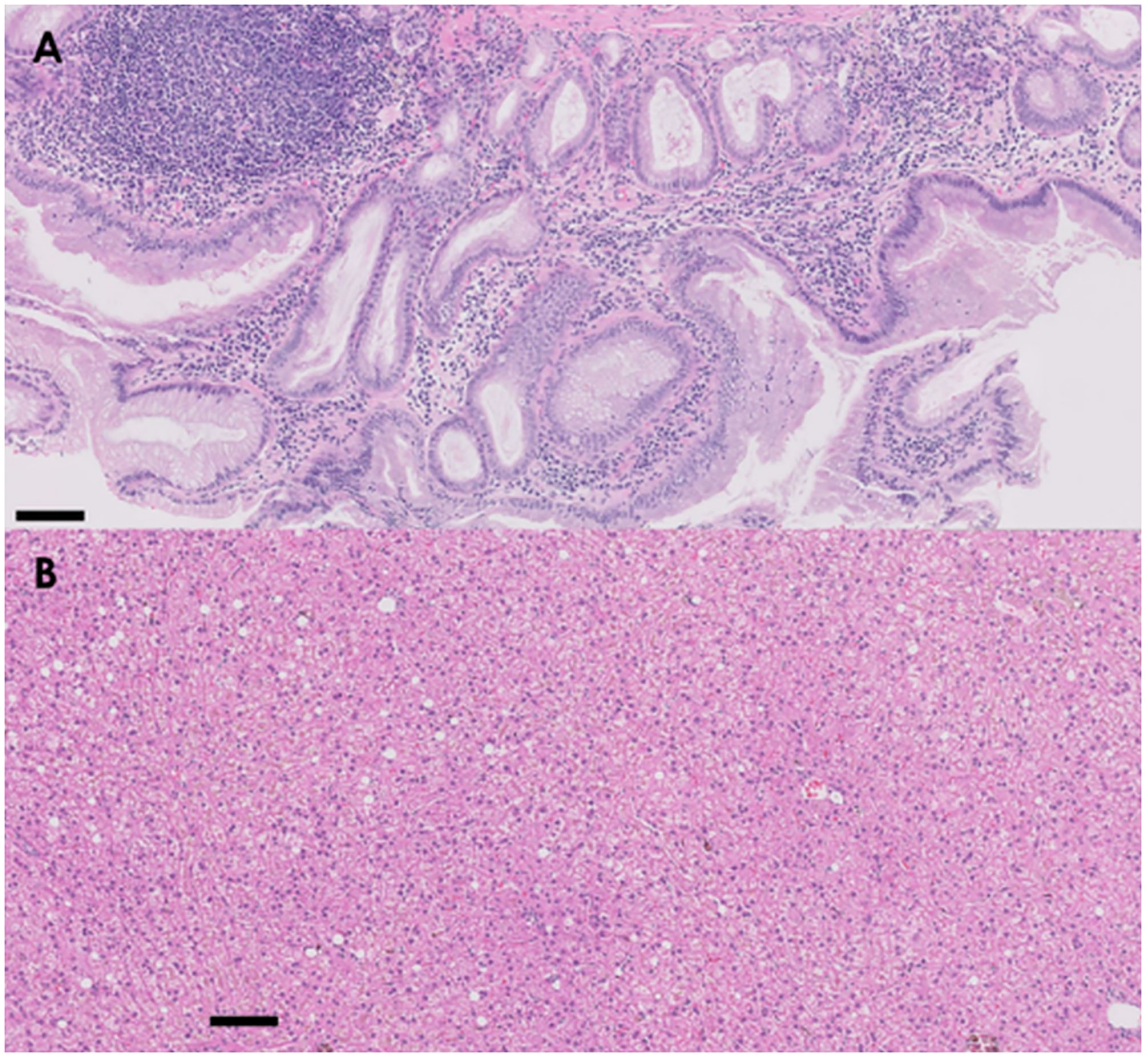
Histology of proliferated GB wall **(A)** and biopsied liver **(B)**. **(A)** The submucosa of the GB is expanded with moderate and diffuse aggregates of lymphocytes and plasma cells. Diffusely expanding the mucosa are multifocal gland structures that are markedly dilated with abundant lightly basophilic mucoid secretions. **(B)** In the liver, there is diffuse and mild swelling of hepatocytes within the centrilobular, midzonal, and portal zones (hematoxylin and eosin staining, 100 × magnification, scale bar: 10 μm).

There were no remarkable findings except for splenomegaly in the chest (four radiographic views) and abdominal radiography (right lateral and ventrodorsal views) (HF-525PLUS, Ecoray, Seoul, Korea). On abdominal ultrasonography (Aplio 300, Canon Medical System, Europe B.V., Zoetermeer, Netherlands), mass-like thickening with irregular margins protruding into the lumen was noted, affecting large portions of the GB, particularly in the region of the neck. These were more echogenic than the contents of the GB lumen, which comprised some anechoic fluid surrounding a rounded organized content that is slightly more echogenic (Figure 1A). Additionally, hypoechoic splenic nodules were observed. Subsequently, CEUS of the GB was performed using SonoVue^®^ (Bracco, Milan, Italy) at a dosage of 0.03 ml/kg. The wash-in for the protruding structures within the GB occurred 9 s after contrast medium (CM) injection. Maximum contrast enhancement (CE) was observed 13 s after CM injection (Figure 1B, left panel). Wash-out occurred 55 s after CM injection. One week later, dynamic CT scan was performed on the GB using a 16-row multi-detector CT scanner (Alexion, TSX-034A, Canon medical system Europe B.V., Zoetermeer, Netherlands) under general anesthesia with isoflurane. As contrast agent, iohexol (Omnipaque 300, GE Healthcare, Shanghai, China) was used at a dosage of 750 mg/kg. CT scan was taken in the following order: pre-contrast CT scan, dynamic CT scan on GB (imaging during 0–34 s after CM administration, four cross-sections), portal phase CT scan (imaging starts 40 s after CM administration, whole body), and delayed phase CT scan (imaging starts 140 s after CM administration, whole body) (kV: 100, mAs: 120, slice thickness: 1 mm). In pre-contrast images, the Hounsfield unit (HU) of the protruding structures within the GB was ~30. Wash-in was confirmed for that structure (HU: 50) more than 20 s after CM injection. More intense and heterogeneous CE was confirmed 34 s after CM injection (HU: 160–270). The dynamic CT scan was stopped due to prolonged breath-holding, and portal and delayed phase CT scans were then performed. CE was confirmed to weaken over time; HU was 120 after 58 s since CM injection and 107 after 159 s (Figure 2). As per diagnostic imaging, vascularized GB polyps were considered. Differential diagnoses for this lesion included inflammatory polyps, mucosal hyperplasia, or neoplasia (adenoma, adenocarcinoma, and carcinoid *in situ*) (4–8). Laparotomy and cholecystectomy were performed considering the fact that the possibility that the lesion observed in the GB was neoplasia could not be excluded, and in human medicine, that asymptomatic gangrenous cholecystitis or emphysematous cholecystitis, which can be life-threatening, may occur. The bile was collected aseptically after the CT scans. At the time of surgery, there were no abnormal findings in the abdomen, including GB rupture or bile leakage. After GB incision, viscous substances and cauliflower-like sessile polyps were confirmed around the cystic duct (Figure 3A). Bile cytology revealed a number of bacilli (Figure 3B). GB wall was collected and fixed in 10% buffered formalin for histopathological analysis. No gross lesions were found in the liver; therefore, a biopsy was performed at one random site in the quadrate lobe. Histopathological examination of the GB revealed chronic lymphoplasmacytic cholecystitis and mucocele (Figure 4A). Additionally, mildly vacuolated hepatocytes were observed in the liver (Figure 4B). *Escherichia coli* was identified as a result of bile culture. The splenic nodules were diagnosed as benign nodular hyperplasia using fine needle aspiration.

The patient was discharged after 1 week of postoperative management. Antibiotics were prescribed for cultured biliary *E*. *coli* for 1 week: amoxycillin-clavulanic acid (13.75 mg/kg, twice daily) and metronidazole (15 mg/kg, twice daily). Except for mild increase in liver enzyme levels immediately after liver biopsy, no abnormalities were observed on physical examination, laboratory blood tests, or ultrasonography for 3 months.

## Discussion

In dogs, echogenic contents within the dog's GB are often diagnosed as sludge or mucocele (9). However, in this case, hyperechoic mass-like protruding structures were observed, which were distinguished from the rounded echogenic organized contents. Therefore, it was necessary to ascertain whether this structure was a tissue receiving blood flow or if it was a sludge or mucocele. Consequently, lack of CE of the GB wall, or a finding of rupture or necrosis, was not observed in CEUS (10). However, GB neoplasia was possible because CE of the protruding structure of the GB was observed in arterial phase (4, 5); it was necessary to determine whether the mass invaded into the adjacent tissues around the GB such as liver, or had metastasized to other organs (11). If the CT revealed findings including infiltration of the mass to the organs outside the GB, extensive resection and biopsy of insulted organs would have been necessary. However, in the arterial phase of the dynamic CT scan, the GB wall and mass-like structures of the GB were clearly distinguished from the surrounding tissue, and therefore, the possibility of invasion into the adjacent tissue such as liver or mesenteric fat was considered low. In the portal and delayed phase, this distinction was unclear, but there were no suspicious findings of metastasis; thus, only the GB was resected.

In the previous literature characterizing imaging features of cholecystitis through CT, it is explained that in acute biliary inflammation, a thickened GB wall and bile ducts are enhanced in the late arterial phase, while in chronic biliary inflammation, a thickened or mineralized GB wall is enhanced in delayed phase (12). Additionally, neoplastic lesions can show strong CE in arterial phase in dogs (5, 13); there is also a report that human adenocarcinoma shows strong CE in the arterial phase in CEUS (4). GB adenoma is reported in a human with cauliflower-like sessile polyps (3). However, herein, the GB sessile polyps showed clear CE in arterial phase on CEUS and dynamic CT scans, but were diagnosed as chronic cholecystitis upon histopathological examination, not as acute inflammation or neoplasia. Thus, chronic cholecystitis should be included in the differential diagnosis, if arterial CE with GB polyps protruding toward the lumen is confirmed. It was also reported that GB mucocele has a significantly higher HU value than GB sludge (14). However, herein, GB contents had low HU close to sludge, but as a result of histopathology, it was diagnosed as GB mucocele. This false negative was because although GB mucocele has a high HU value, the deviation is very large, and the HU value between sludge and mucocele has a large overlap (14).

CE of the GB sessile polyps was observed using two modalities. CEUS is more advantageous than dynamic CT as the lesion can be imaged without anesthesia. Additionally, it took longer in CT for CE of the lesion, presumably because cardiovascular function was suppressed by isoflurane (15). When dynamic CT scan was performed, the heart rate decreased from 80 to 46 beats per minute (BPM) during anesthesia. However, there was no bradyarrhythmia, and heart rate increased to 120 BPM after atropine administration before surgery. Another limitation of dynamic CT was that the motion artifact due to diaphragm movement had to be reduced through breath control, which stopped exhalation for an extended time period. However, unlike CEUS, which evaluates one cross-section, dynamic CT has the advantage of simultaneously evaluating multiple cross-sections and can evaluate wide area around the GB. CT also has the advantage of being able to evaluate metastases in the whole body.

Herein, chronic cholecystitis did not cause clinical signs, and blood tests were normal except for leukocytopenia. In humans, there are case reports of asymptomatic gangrenous cholecystitis and emphysematous cholecystitis (16, 17). However, CEUS and dynamic CT provide good imaging for the GB sessile polyps; thus, they could help diagnose asymptomatic polypoid chronic cholecystitis.

*Escherichia coli* was detected in the bile, and the patient had practiced coprophagia for several years. To the best of our knowledge, no studies have clearly established the relationship between coprophagia and chronic cholecystitis. Additionally, the etiology of bacterial cholecystitis and its correlation with cholangitis are not clearly established (1). However, there are studies suggesting that ascending infection from the gastrointestinal tract (GIT) and hematogenous infection from sepsis cause bacterial cholecystitis (1, 18). Fecal pathogens can infect the dog through coprophagia (19). Moreover, there was a case report wherein *Cyniclomyces guttulatus* was cultured in the bile of a dog with cholecystitis (2). This yeast is a normal flora of rabbit GIT, and coprophagia preserves it (20). It can also cause opportunistic infections in the GIT of dogs ingesting rabbit feces (20). Because she had no history of diseases including sepsis, ascending *E. coli* infection from the GIT is the likely cause of her chronic cholecystitis. The possibility that coprophagia may have contributed to this infection cannot be completely excluded, however.

The patient had mild leukocytopenia of unknown cause, which may have caused cholecystitis. Although dogs with neutropenia may not display obvious clinical signs, they are known to be susceptible to bacterial and fungal infections (21).

The liver showed mild vacuolar degeneration. Considering that cholecystitis may be associated with decreased GB contractility [(22), #17], vacuolar hepatopathy could be caused by cholecystitis. However, vacuolar hepatopathy is a non-specific change in the liver, and may not be clinically significant. This change can be caused by diabetes mellitus, hypothyroidism, hyperadrenocorticism, and stress conditions (23–25); laboratory blood and urine tests ruled out the above endocrinopathy in this case.

This study has several limitations. First, wash-out of the CM could not be observed on dynamic CT due to prolonged breath-holding. A method for reducing motion artifacts using the predictive respiratory gating system in humans is studied (26); however, to the best of our knowledge, there are no studies on GB imaging using this technique in dogs; further studies are needed to overcome these artifacts. Second, the etiology for this patient is incompletely elucidated. The dog had no history of systemic disease; thus, an ascending infection of *E. coli* from the GIT might have caused cholecystitis. However, we cannot clarify whether coprophagia was involved in the occurrence of chronic cholecystitis; further investigations are warranted.

In conclusion, chronic cholecystitis should be considered when contrast-enhanced structures protruding toward the GB lumen are confirmed through CEUS or dynamic CT scan in arterial phase. In this regard, CEUS has the advantage of imaging the lesion without anesthesia, whereas dynamic CT has the advantage of evaluating a wider area including the gallbladder and adjacent structures. This is expected to help in planning the surgical site and selecting the target organ for biopsy. Since both modalities image lesions well, a more useful modality can be selected and used in consideration of various circumstances, including the patient's condition. Additionally, sessile polyps caused by chronic cholecystitis can mimic neoplasia and acute cholecystitis, which can cause GB wall thickening or CE in arterial phase (3, 5, 12). Therefore, it appears that histology is necessary for differential diagnosis.

## Data Availability Statement

The original contributions presented in the study are included in the article/supplementary material, further inquiries can be directed to the corresponding author/s.

## Ethics Statement

Ethical review and approval was not required for the animal study because, this study is a case report of examinations, surgeries, and biopsies performed for the purpose of treatment of patients, and no action contrary to treatment was performed. Written informed consent was obtained from the owners for the participation of their animals in this study.

## Author Contributions

JL and HY: conception and design and drafting the article. JL, JK, SH, and HY: acquisition of data. JL, JK, SH, KL, and HY: analysis and interpretation of data, revising article for intellectual content, and final approval of the completed article. All authors contributed to the article and approved the submitted version.

## Funding

The research was supported by Basic Science Research Program through the National Research Foundation of Korea (NRF) funded by the Ministry of Education (2019R1A6A1A03033084).

## Conflict of Interest

The authors declare that the research was conducted in the absence of any commercial or financial relationships that could be construed as a potential conflict of interest.

## Publisher's Note

All claims expressed in this article are solely those of the authors and do not necessarily represent those of their affiliated organizations, or those of the publisher, the editors and the reviewers. Any product that may be evaluated in this article, or claim that may be made by its manufacturer, is not guaranteed or endorsed by the publisher.

## Abbreviations

GB: gallbladder
CEUS: contrast-enhanced ultrasonography
CT: computed tomography
CM: contrast medium
CE: contrast enhancement
*E. coli*: *Escherichia coli*.
